# Development of loop-mediated isothermal amplification assay for specific and rapid detection of differential goat Pox virus and Sheep Pox virus

**DOI:** 10.1186/1471-2180-14-10

**Published:** 2014-01-17

**Authors:** Zhixun Zhao, Bin Fan, Guohua Wu, Xinmin Yan, Yingguo Li, Xiaoli Zhou, Hua Yue, Xueling Dai, Haixia Zhu, Bo Tian, Jian Li, Qiang Zhang

**Affiliations:** 1 Key Laboratory of Animal virology of the Ministry of Agriculture, State Key Laboratory of Veterinary Etiological Biology, Lanzhou Veterinary Research Institute, CAAS, Lanzhou, Gansu, PR China; 2 Chongqing entry exit inspection and quarantine bureau, Chongqing, PR China; 3 Yunnan entry exit inspection and quarantine bureau, Kunming, PR China; 4 College of Life Science and Technology, Southwest University for Nationalities, Chengdu, PR China; 5 Key Laboratory of Desert and Desertification, Cold and Arid Regions Environmental and Engineering Research Institute, CAS, Lanzhou, Gansu, PR China

**Keywords:** Goat pox virus (GTPV), Sheep pox virus (SPPV), Inverted terminal repeat (ITR) regions, Loop-mediated isothermal amplification (LAMP), Differential diagnosis

## Abstract

**Background:**

Capripox viruses are economically important pathogens in goat and sheep producing areas of the world, with specific focus on goat pox virus (GTPV), sheep pox virus (SPPV) and the Lumpy Skin Disease virus (LSDV). Clinically, sheep pox and goat pox have the same symptoms and cannot be distinguished serologically. This presents a real need for a rapid, inexpensive, and easy to operate and maintain genotyping tool to facilitate accurate disease diagnosis and surveillance for better management of Capripox outbreaks.

**Results:**

A LAMP method was developed for the specific differential detection of GTPV and SPPV using three sets of LAMP primers designed on the basis of ITR sequences. Reactions were performed at 62°C for either 45 or 60 min, and specificity confirmed by successful differential detection of several GTPV and SPPV isolates. No cross reactivity with *Orf* virus, foot-and-mouth disease virus (FMDV), *A. marginale Lushi* isolate*, Mycoplasma mycoides subsp. capri, Chlamydophila psittaci, Theileria ovis, T. luwenshuni, T. uilenbergi* or *Babesia sp* was noted*.* RFLP-PCR analysis of 135 preserved epidemic materials revealed 48 samples infected with goat pox and 87 infected with sheep pox, with LAMP test results showing a positive detection for all samples. When utilizing GTPV and SPPV genomic DNA, the universal LAMP primers (GSPV) and GTPV LAMP primers displayed a 100% detection rate; while the SPPV LAMP detection rate was 98.8%, consistent with the laboratory tested results.

**Conclusions:**

In summary, the three sets of LAMP primers when combined provide an analytically robust method able to fully distinguish between GTPV and SPPV. The presented LAMP method provides a specific, sensitive and rapid diagnostic tool for the distinction of GTPV and SPPV infections, with the potential to be standardized as a detection method for Capripox viruses in endemic areas.

## Background

Sheep pox and goat pox are economically important diseases in goat and sheep producing areas of the world. Sheep pox and goat pox result from infection by SPPV or GTPV respectively, and are closely related members of the *Capripoxvirus* genus in the family *Poxviridae*. Clinically, sheep pox and goat pox have indistinguishable symptoms. Several PCR-based assays have been reported to distinguish SPPV from GTPV including cleaved amplification polymorphism sequence-tagged sites (RFLP-PCR) and real-time PCR (qPCR) [1-5]. In our laboratory, distinction of GTPV from SPPV was established via a *Hinf* I digest of the p32 gene, followed by a sequence alinment of G-protein-coupled chemokine receptor (GpCR) genes [5,6]. Some of the advantages of qPCR include speed, sensitivity, and real time monitoring to determine exact concentrations. However, this approach requires expensive high precision instrumentation and specialized training for operation and data analysis, presenting a need for a more convenient alternative that is robust, inexpensive, and easy to operate and maintain.

Recently, loop-mediated isothermal amplification (LAMP) has been developed for the diagnosis of a number of diseases [1,7,8]. The LAMP reaction can be conducted under isothermal conditions ranging 60-65°C by using four or six primers recognizing six or eight distinct regions [9]. LAMP produces large quantities of amplified product resulting in easy visual detection either via turbidity or fluorescence [10]. The present study established the ability of LAMP assays to differential detect GTPV and SPPV through the targeting of inverted terminal repeat (ITR) sequences. Compared to conventional PCR techniques, the newly established LAMP assay is simple, efficient, cost-effective and convenient, making it a useful diagnostic tool for clinical samples.

## Results

### Primers and gene sequences

**Figure 1:**
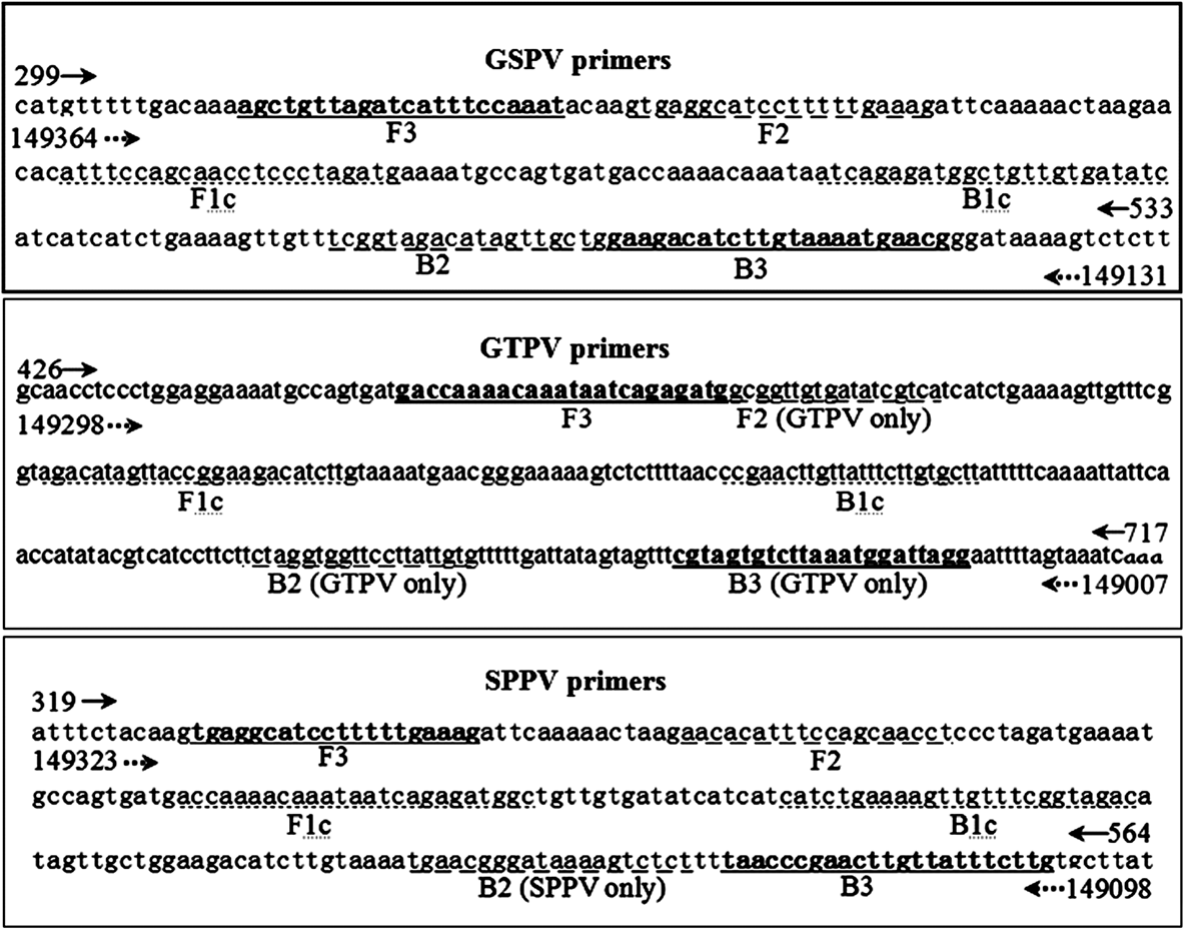
**Target gene sequences and primers.** Nucleotide sequences of the LAMP amplicon (ITR, GenBank accession no. AY077834.1 for GSPV and SPPV primers, and GenBank accession no. AY077836.1 for GTPV primers) and locations of the LAMP primers along the sequence. Forward and reverse ITR amplicon are indicated by solid line arrows () and dashed line arrows (), respectively.

Several GTPV and SPPV genomic sequences were downloaded from GenBank and aligned using MegAlign, with the most conserved ITR segments selected as targets. All LAMP primers were designed using an online software (http://primerexplorer.jp/elamp3.0.0/index.html; Eiken Chemical Co., Ltd., Tokyo, Japan), with four primers designed for the LAMP assay (Figure 1; Table 1). These included two outer primers (F3 and B3), a forward inner primer FIP (F1c - F2) and a backward inner primer BIP (B1c - B2).

**Table 1:** Primer sets designed to detect goat pox and sheep pox virus by LAMP and universal LAMP primers designed for GTPV and SPPV

**Primers name**	**Each set of primer**	**Type**	**Length**	**Sequence(5’-3’)**	**Notes**
GSPV primers	GSF3	Forward outer	22	AGCTGTTAGATCATTTCCAAAT	The universal lamp primers for GTPV and SPPV, the predicted length of Lamp is 204 bp.
	GSB3	Backward outer	23	CGTTCATTTTACAAGATGTCTTC	
	GSFIP	Forward inner primer (F1c + F2)	44	CATCTAGGGAGGTTGCTGGAAAT	
				-GTGAGGCATCCTTTTTGAAAG	
	GSBIP	Backward inner primer (B1 + B2c)	43	ATCAGAGATGGCTGTTGTGATATC	
				-CAGCAACTATGTCTACCGA	
GTPV primers	GF3	Forward outer	24	ACCAAAACAAATAATCAGAGATG	The special lamp primers for GTPV, the predicted length of Lamp is 245 bp. The underlined sequences match specifically for GTPV genome but not SPPV genome.
	GB3	Backward outer	23	*CCTAATCCATTTAAGACACTACG*	
	GFIP	Forward inner primer (F1c + F2)	43	AAGATGTCTTCCGGTAACTATGTCT	
				-*GCGGTTGTGATATCGTCA*	
	GBIP	Backward inner primer (B1 + B2c)	45	CCGAACTTGTTATTTCTTGTGCTT	
				-*CACAATAAGGAACCACCTAGA*	
SPPV primers	SF3	Forward outer	20	TGAGGCATCCTTTTTGAAAG	The special lamp primers for SPPV, the predicted length of Lamp is 215 bp. The underlined sequence match specifically for SPPV genome but not GTPV genome.
	SB3	Backward outer	22	AAGAAATAACAAGTTCGGGTTA	
	SFIP	Forward inner primer (F1c + F2)	44	GCCATCTCTGATTATTTGTTTTGGT	
				-*AACACATTTCCAGCAACCT*	
	SBIP	Backward inner primer (B1 + B2c)	45	CATCTGAAAAGTTGTTTCGGTAGAC	
				-AGAGACTTTTATCCCGTTCA	

### Reaction condition optimization for GTPV and SPPV detection by LAMP

**Figure 2:**
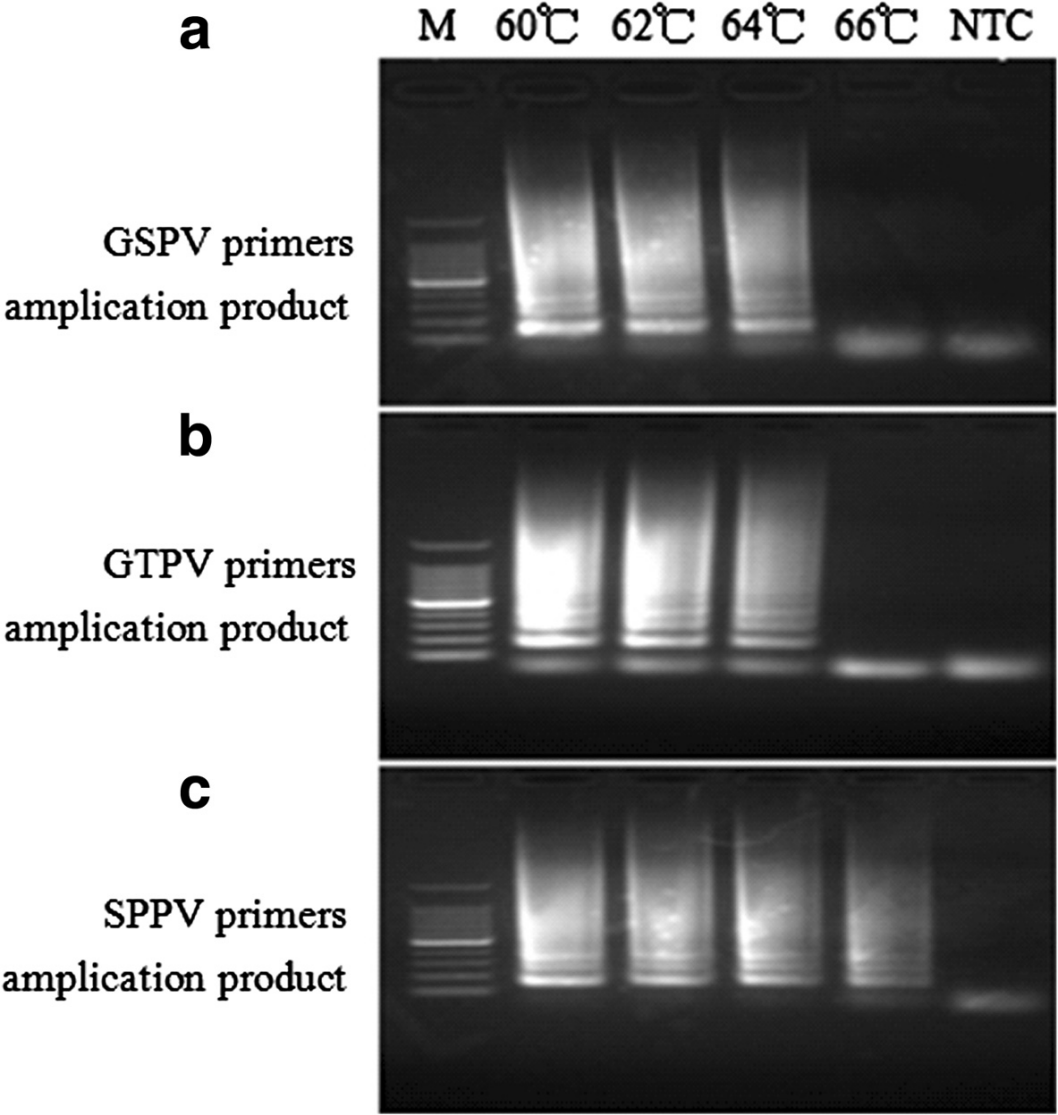
**Optimization of incubation temperature for LAMP reaction in the detection of GTPV or SPPV using different primer sets.** Agarose gel electrophoresis showing the effect of temperature on LAMP reaction. **(a)** GSPV primer amplification products using 100 ng SPPV gDNA as template. **(b)** GTPV primer amplification products using 100 ng GTPV gDNA as template. **(c)** SPPV primer amplification products using 100 ng SPPV gDNA as template. Lane M:100 bp DNA Ladder Marker (TaKaRa, Dalian) and no template control (NTC).

To determine optimal reaction temperatures for each LAMP primer set, the SPPV genome was used as a template for the GSPV and SPPV primer sets and the GTPV genome used for the GTPV primer set. Reaction temperatures were altered to include 60°C, 62°C, 64°C and 66°C for 60 min, followed by a 80°C heating for 2 min. Two microliters of each LAMP product was examined via gel electrophoresis and imaged. The results showed the GSPV and GTPV primer sets could successfully amplify the target gene at all experimental temperature levels, with the exception of 66°C (Figure 2a, 2b), while the SPPV primers successfully amplified the target gene at all experimental temperature levels (Figure 2c).

**Figure 3:**
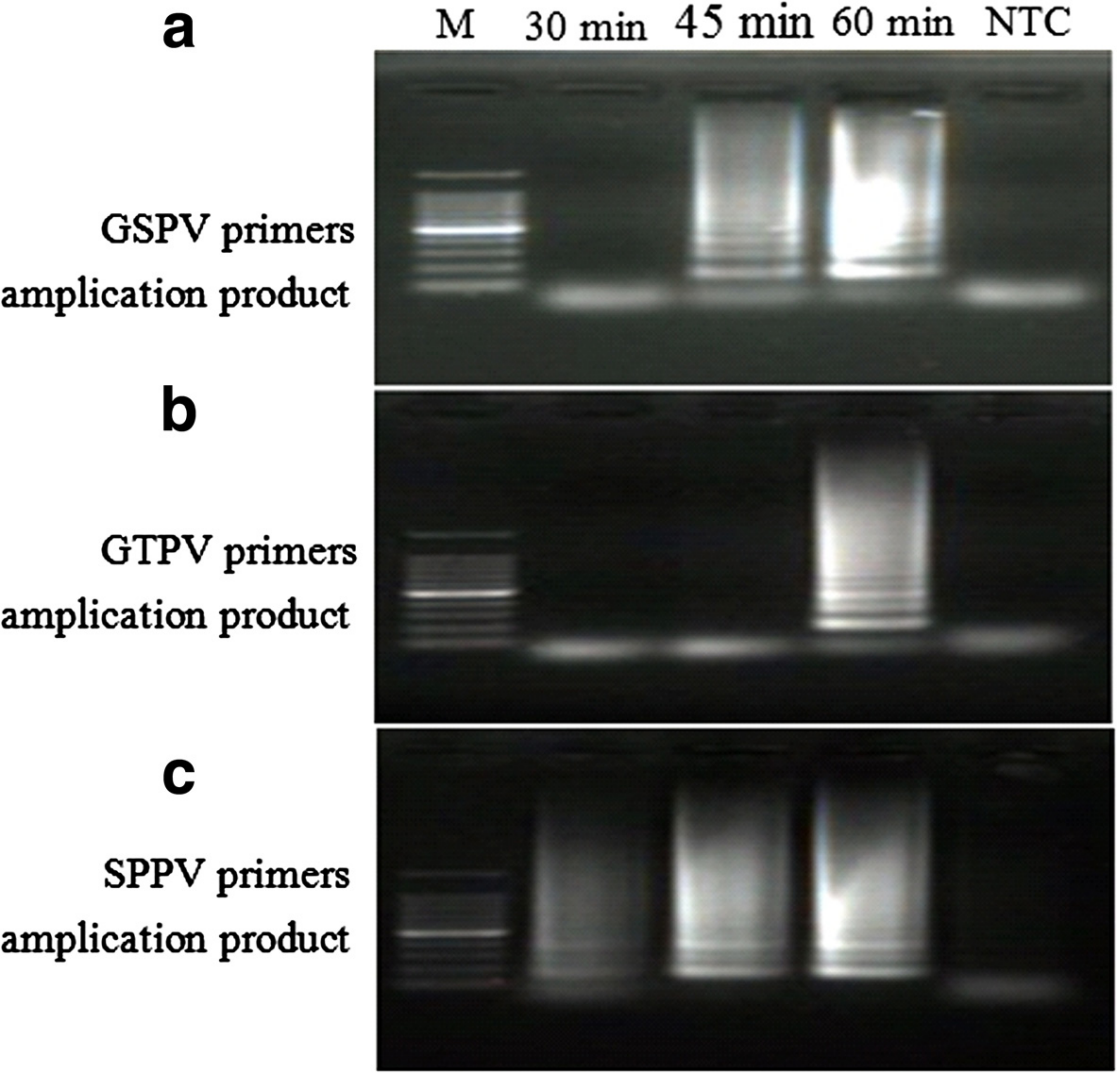
**Optimization of incubation time for LAMP reaction in the detection of GTPV or SPPV using different primer sets.** Agarose gel electrophoresis showing the effect of time on LAMP reaction. **(a)** GSPV primer amplification products using 100 ng SPPV gDNA as template. **(b)** GTPV primer amplification products using 100 ng GTPV gDNA as template. **(c)** SPPV primer amplification products using 100 ng SPPV genome DNA as template. Lane M: 100 bp DNA Ladder Marker (TaKaRa, Dalian) and no template control (NTC).

When attempting to optimize incubation time, GSPV primers at 62°C were able to amplify the target gene following a 45 min or 60 min incubation, but unable to display successful amplification following a 30 min incubation (Figure 3a). When examining GTPV primers at 62°C, only an incubation time of 60 min resulted in successful amplification (Figure 3b), while the SPPV primers displayed successful amplification during all experimental incubation periods (Figure 3c).

### LAMP and PCR sensitivity for detection of GTPV and SPPV

To determine the reaction sensitivity of each LAMP primer set, SPPV gDNA (genomic DNA) was used as a template for the GSPV and SPPV primer sets, while GTPV gDNA was used as templates for the GTPV primer set. All template concentrations were established via nucleic acid meter measurement and the copy number calculated. SPPV gDNA was serially diluted to achieve 1.037 × 10^9^ - 1.037 × 10^0^ copies of template, while GTPV gDNA was serially diluted to achieve 1.045 × 10^9^ - 1.045 × 10^0^ copies of template. The reaction was carried out at 62°C for 45 min or 60 min, followed by a 2 min incubation at 80°C. Two microliters of each LAMP product was analyzed via gel electrophoresis and UV imaged.

**Figure 4:**
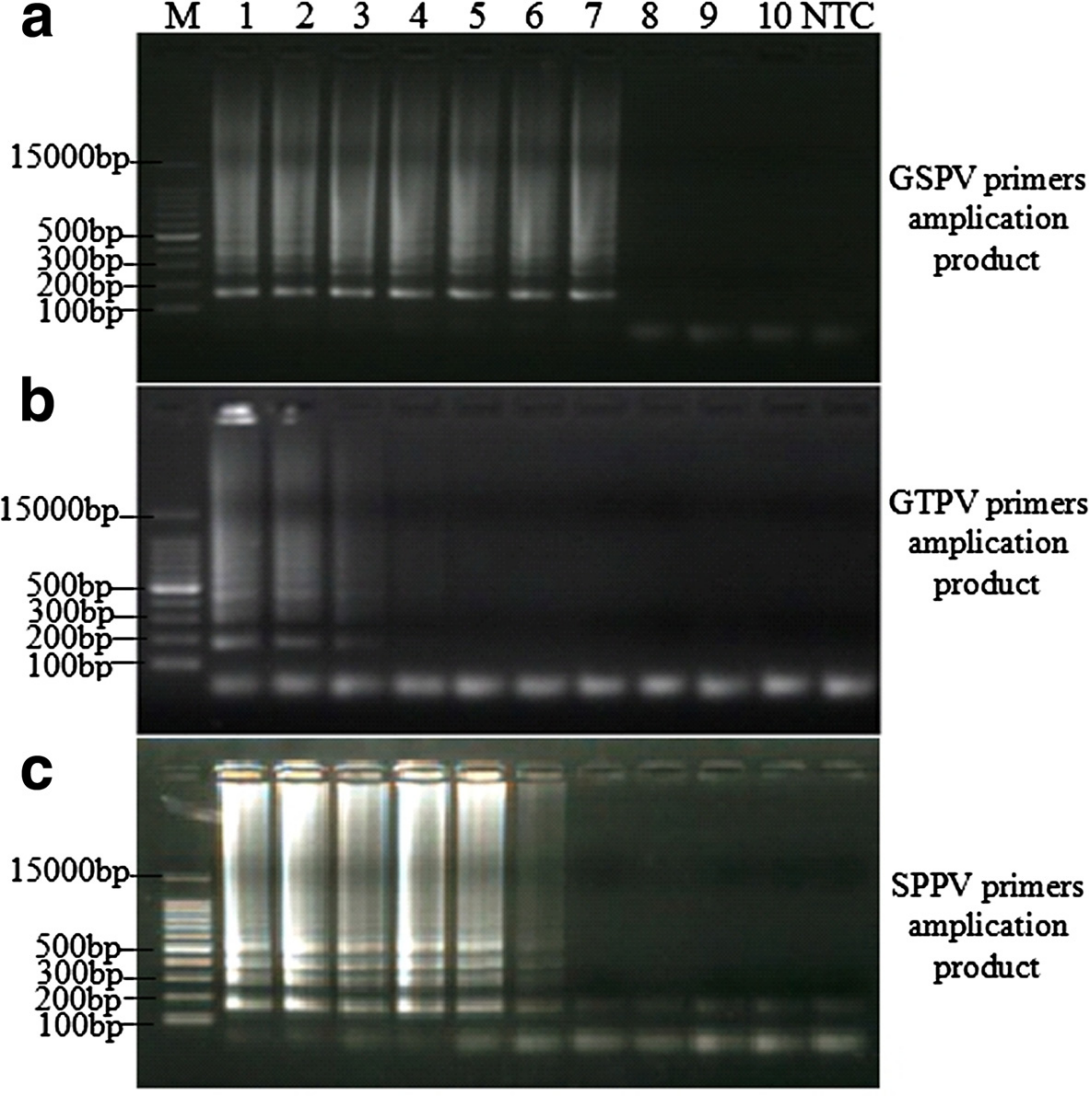
**LAMP sensitivity.** Amplification using serially diluated template followed by electrophoresis. **(a)** and **(c)** lane 1–10: SPPV gDNA serially diluted samples (1.037 × 10^9^–1.037 × 100 copies of template), **(b)** lane 1–10: GTPV gDNA serially diluted samples (1.045 × 10^9^ - 1.045 × 10^0^ copies of template). Lane M: 100 bp DNA Ladder Marker (TaKaRa, Dalian) and no template control (NTC).

Electrophoretic analysis of the amplification of varying template concentrations incubated for 45 min showed successful amplification when using GSPV primers in conjunction with template copy numbers between 1.037 × 10^9^ - 1.037 × 10^3^, while the control sample showed no banding (Figure 4a). We can see 1.037 × 10^3^ copies of the template be detected with general lamp GSPV primers at 62°C, after amplification 45 min. When incubating for 60 min, GTPV primers were able to amplify specific products with DNA template copy numbers between 1.045 × 10^9^ - 1.045 × 10^6^, while the control group showed no banding (Figure 4b) and lower DNA template concentrations virtually undetectable. When examining amplification levels using SPPV primers after incubation for 45 min, (Figure 4c) amplification product was noted with DNA template copy numbers between 1.037 × 10^9^ - 1.037 × 10^4^, while the control group showed no banding.

The detection sensitivity of reactions with different incubation temperatures and incubation times for each primer set showed in Table 2.

**Table 2:** The detection sensitivity of reactions with different incubation temperatures and incubation times for each primer set

**Sensitivity of reactions**	**Temperature option (incubation 60 min) (°C)**				**Time option (incubation at 60°C) (min)**			**Sensitivity (incubation at 60°C for 60 min) (copies/reaction)**									
	**60**	**62**	**64**	**66**	**30**	**45**	**60**	**10**^**9**^	**10**^**8**^	**10**^**7**^	**10**^**6**^	**10**^**5**^	**10**^**4**^	**10**^**3**^	**10**^**2**^	**10**^**1**^	**10°**
GSPV Primer	**+**	**+**	**+**	**-**	**-**	**+**	**+**	**+**	**+**	**+**	**+**	**+**	**+**	**+**	**-**	**-**	**-**
GTPV Primer	**+**	**+**	**+**	**-**	**-**	**-**	**+**	**+**	**+**	**+**	**+**	**-**	**-**	**-**	**-**	**-**	**-**
SPPV Primer	**+**	**+**	**+**	**+**	**+**	**+**	**+**	**+**	**+**	**+**	**+**	**+**	**+**	**-**	**-**	**-**	**-**

Note: Optimization of incubation temperature and time for LAMP reactions when detecting about 100 ng of GTPV or SPPV genomic DNA using different primer sets. “+” stand for positive result and “-” stand for negative result.

### LAMP assay specificity

Reaction specificity was determined for each LAMP primer set utilizing GTPV, SPPV, Orf virus, FMDV, *M. ovipneumoniae*, *Chlamydia psittaci*, *L. interrogans*, *Toxoplasma gondii*, *Theileria* and *Babesia sp* templates. Reactions were carried out at 62°C, while utilizing the optimized incubation time of 45 min for GSPV and SPPV primers and 60 min for GTPV primers, followed by a 2 min incubation at 80°C. Two microliters of each LAMP product was analyzed via gel electrophoresis and UV imaging.

**Figure 5:**
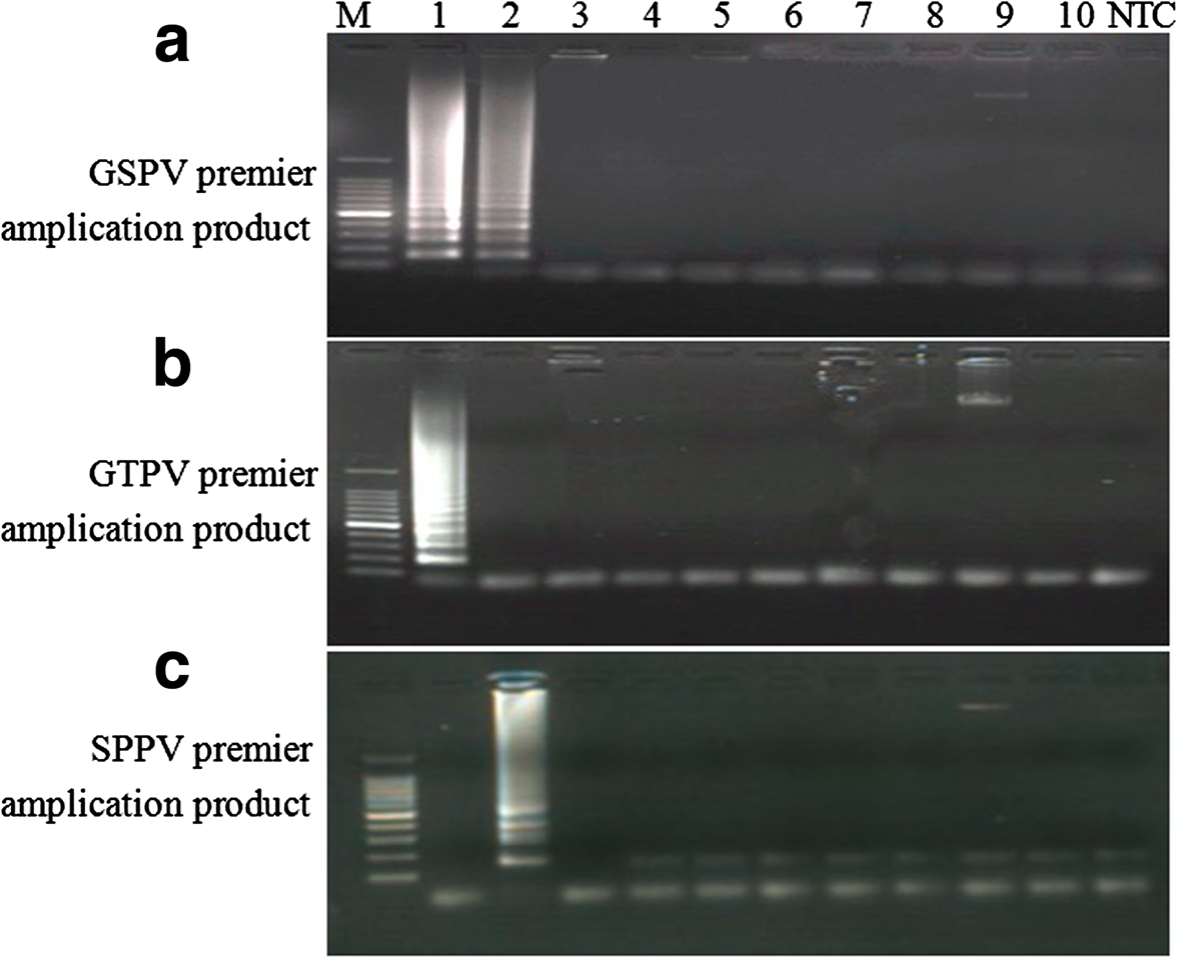
**Specificity of LAMP for detection of different pathogenic nucleic acids using different primer sets.** Approximately 100 ng of DNA or cDNA template from ten different sheep or goat pathogens was used in LAMP reaction. **(a)** GSPV primers amplification products. **(b)** GTPV primer amplification products. **(c)** SPPV primer amplification products. Agarose gel electrophoresis (2.5%) of LAMP products stained with ethidium bromide and visualized under a UV transilluminator. Lane 1: GTPV; Lane 2: SPPV; Lane 3: *Orf* virus; Lane 4: FMDV O/China99; Lane 5: *M. ovipneumoniae*; Lane 6: *Chlamydia psittaci*; Lane 7: *L.interrogans*; Lane 8: *Toxoplasma gondii*; Lane 9:Babesia sp; Lane 10: Theileria; C: no template control (NTC) and Lane M: 100 bp DNA Ladder Marker (TaKaRa, Dalian).

The specificity of the LAMP assay results reveal that the GSPV primers could amplify the target gene in SPPV and GTPV gDNA, but were unable to successfully amplify Orf, FMDV, *M. ovipneumoniae*, *Chlamydia psittaci*, *L. interrogans*, *Toxoplasma gondii*, *Babesia sp*, *Theileria* and the negative control sample (Figure 5a). GTPV primers were able to amplify the target gene in GTPV gDNA when incubated at 62°C for 60 min, while showing unsuccessful amplification of other pathogenic genomes (Figure 5b). Meanwhile, SPPV primers could amplify the target gene in SPPV gDNA when incubated at 62°C for 45 min, while showing unsuccessful amplification of other genomic templates (Figure 5c). However, all three sets of primers could amplify a large gene in the *Babesia sp* gDNA (Figure 5), but luckily this has no effect on the assay results. To further confirm the specificity of the amplified ITR sequences, LAMP products were sequenced. Proceeding bioinformatic analysis, all LAMP amplicons displayed 100% sequence identity to their corresponding ITR sequences (data not shown).

### Evaluation of the LAMP assay

One hundred thirty-five preserved epidemic materials were evaluated to revealed 48 samples infected with goat pox and 87 infected with sheep pox (Table 3). All samples were assessed using the GSPV LAMP diagnosis method to yield a 100% detection rate, which was consistent with the laboratory tested results. Samples assessed using the GTPV LAMP diagnosis method also showed a 100% detection rate in agreement with the laboratory tested results. Samples evaluated using the SPPV LAMP method showed 86 positive detection results and one negative, to yield a detection rate of 98.8%.

**Table 3:** Results of LAMP detection with clinic samples

**Methods**	**Sample size**	**Positive result**	**Negative result**	**Positive detection rates (%)**	**Sample category**
GSPV lamp detection	135	135	0	100	48 GTPV samples and 87 SPPV samples
GTPV lamp detection	135	48	0	100	
SPPV lamp detection	135	86	1	98.8	

## Discussion

GTPV and SPPV contain double-stranded DNA genomes that are approximately 150 kbp and share at least 147 putative genes, to include conserved poxvirus replicative and structural genes and genes likely involved in virulence and host range [11]. Restriction endonuclease analysis and cross-hybridization studies of SPPV and GTPV indicate that these viruses, although closely related (estimated 96 to 97% nucleotide identity), can be distinguished from one another and may undergo recombination in nature [11-14]. Several PCR tests have been developed for the detection of *Capripoxviruses*[5,6,15-24]. In our laboratory, distinction of GTPV from SPPV was established via a *Hinf* I digest of the p32 gene, followed by sequence alinment of GpCR genes [5,6]. However, these methodologies are time consuming, expensive and require experienced laboratory staff. This presents a real need for a more convenient alternative to PCR that is robust, inexpensive, and easy to operate and maintain.

LAMP is a novel nucleic acid isothermal amplification technique developed by Notomi [25] and serves as a powerful gene amplification tool due to its high specificity and sensitivity under isothermal condition [26-28]. Previous LAMP methods were developed for the rapid detection of *Capripoxviruses*[1] and were unable to distinguish SPPV from GTPV. The present study aimed to develop a LAMP method for the rapid distinction of SPPV from GTPV, and to evaluate its applicability through field sample testing. LAMP primer design was based on six regions in the target sequence designated from the 5’-end as F3, F2, F1, B1, B2, and B3 (Figure 1). The forward inner primer (FIP) consists of the F2 sequence (at its 3’ end) that is complementary to the F2c region and the same sequence as the F1c region at its 5’ end. The four key factors in the LAMP primer design are the T_m_, primer end stability, GC content and secondary structure. T_m_ is estimated using the Nearest-Neighbor method, which is an approximation method that provides values closest to the actual values. The T_m_ for each region was determined to be ~ 65°C (64 - 66°C) for F1c and B1c, ~ 60°C (59 - 61°C) for F2, B2, F3, and B3 and ~ 60°C for the loop primers. Since primers serves as the starting point of DNA synthesis, a certain degree of stability must be achieved. The 3’ ends of F2/B2, F3/B3, and LF/LB and the 5’ end of F1c/B1c were designed to have a free energy of -4 kcal/ mol or less. The 5’ end of F1c after amplification corresponds to the 3’ end of F1, making its stability important. Primers were designed to have GC content between ~40% to 65%, with 50% to 60% GC content optimal. It is important, particularly for inner primers, that primers are designed to eliminate the formation of secondary structures. Additionally, it is important to prevent primer dimerization by ensuring that the 3’ ends are not complementary.

The most critical aspect of the current study is to design robust primers able to achieve differential detection of SPPV and GTPV, thus warranting a rigorous design process. To optimize SPPV primer specificity, primers comparison analysis shows that B2 of the SPPV primers (that is the composition of SPPV BIP) is a characteristic sequence in the SPPV genome (see SPPV SFIP underlined sequence in Table 1) and does not exist in the GTPV genome. The calculated dimer (minimum) dG SPPV LAMP primer was -2.49 kcal/mol, the 3’ ends of F2/B2 and F3/B3 and the 5’ end of F1c/B1c were designed to have a free energy of -4 kcal/mol or less and GC rates were around 0.4. The calculated T_m_ for F3 was 55.67 and for B3 was 55.34, making both of the T_m_s relatively close. Additionally, the calculated T_m_ for F1c was 60.08 and for B1c was 60.10, yielding very close T_m_s for both. Lastly, the calculated T_m_ for F2 was 56.96 and for B2 was 55.42, again yielding close T_m_s. These indicators are more in line with the general LAMP primer design requirements generating high specificity and sensitivity in theory, and which was experimentally validated. The SPPV primer achieved high sensitivity and specificity in the presence of 1.037 × 10^4^ copies of DNA template.

In order to guarantee GTPV primer specificity, primer sequence specificity was assessed via comparative analysis to shows that B3/F2 of the GTPV primers (that is, the composition of GTPV FIP) and B2 of the GTPV primers (that is the composition of GTPV BIP) are characteristic sequences in the GTPV genome (see GTPV GF3, GFIP and GBIP underlined sequences in Table 1) and does not exist in SPPV genome. The designed GTPV LAMP primers had higher specificity than SPPV primers in theory due to a GC rate around 0.4, the 3’ ends of F2/B2 and F3/B3 and the 5’ end of B1c were designed with a free energy of -4 kcal/mol. The calculated T_m_ for F3 was 56.01 and for B3 was 56.13, making them relatively close. Additionally, the calculated T_m_ for F1c was 61.20 and for B1c was 60.03, also making them relatively close. Lastly, the calculated T_m_ for F2 was 56.21 and for B2 was 56.39, yielding very close T_m_s. These indicators are more in line with the general LAMP primer design requirements. The calculated dimer (minimum) dG GTPV lamp primer was only -1.18 kcal/mol, and 5’ end of F1c had a free energy of only -3.90 kcal/mol, less than the target -4 kcal/mol. These parameters should results in primers with higher specificity but lower sensitivity in theory, which was experimentally validated. The specificity of the SPPV primer was high, but the sensitivity was lower as demonstrated by a need of 1.045 × 10^6^ copies of template.

All GSPV primers were designed to match all sequences characteristics in the GTPV and SPPV genomes, with indicators more in line with general LAMP primer design requirements. The only drawback in the design was the 5’ end of F1c having a free energy of -3.48 kcal/mol, which was less than the ideal -4 kcal/mol, but the predicted high degree of specificity and adequate sensitivity were experimentally validated. The specificity of the GSPV primers was high, in addition to achieving a high sensitivity as demonstrated by the use of 1.037 × 10^3^ copies of template.

Although the predicted SPPV primer specificity could have been higher, experimentation showed its inability to produce an amplification product from other pathogenic genomes, thus confirming the ability of the SPPV primer to specifically detect SPPV alone. While both predictive and experimental evidence displayed a high degree of specificity in GTPV primers, but a lower sensitivity, which can be rectified through its combining with the GSPV primers. In short, combining all three sets of primers enables the quick and efficient detection of GTPV and SPPV. While the methods established in this study are effective, they could be further optimized by designing loop primers to further reduce experimentation time and visualization could be more streamlined through the utilization of fluorescence dyes.

In clinical samples testing found that SPPV LAMP primer detected miss one case which can be detected by GSPV LAMP primer. The possible reason is that all SPPV nucleic acid concentration in the samples were within the scope of SPPV detection sensitivity, but the nucleic acid content of the miss sample was smaller than the SPPV LAMP highest sensitivity(1.037 × 10^4^ copies) but within GSPV LAMP primer detection sensitivity,(only reach to 1.037 × 10^3^ copies). So the judgment of the samples should be to test again, or in other ways for further confirmation, and pay more attention to the concentration of the sample in the process of sample handling.

The presented experimentation has shown that the sequence of the GTPV primer can provide specificity and rapid detection of GTPV nucleic acids, but was unable to detect SPPV nucleic acids under the same conditions. On the other hand, the SPPV primer can provide specificity and rapid detection of SPPV nucleic acids, but was unable to detect GTPV nucleic acids under the same conditions. However, the GSPV primer can rapidly amplify GTPV nucleic acid and SPPV nucleic acid. Collectively, GSPV, GTPV and SPPV LAMP primers when combined possess the analytical ability to fully distinguish between GTPV and SPPV.

## Conclusions

These laboratory studies showed that the LAMP method of differential GTPV and SPPV detection is inexpensive, rapid, simplistic, specific, and sensitive. These attributes make this method an optimal detection system for field detection and differential diagnosis of GTPV and SPPV. It is a promising assay for extensive application and rapid diagnosis of GTPV and SPPV infection in the laboratory and the field, especially in countries that lack the resources needed for molecular diagnostic techniques.

## Methods

### Ethics statement

This study was approved by the Animal Ethics Committee of the Lanzhou Veterinary Research Institute, Chinese Academy of Agricultural Sciences (approval number LVRIAEC 2012–018). Goats and sheep, from which tissues samples were collected, were handled according to good animal practices required by the Animal Ethics Procedures and Guidelines of the People’s Republic of China (AEPGPRC). Tissue collections were performed as part of a routine disease monitoring and surveillance process for these livestock with owner consent.

### Gene sequences and primers design

All LAMP primers were designed using PrimerExplorer v3 software (http://primerexplorer.jp/) available from the Eiken Chemical Company website (http://loopamp.eiken.co.jp/e/index.html). Software settings were adjusted to account for an AT-rich template, to include selecting a lower melting temperature (T_m_), increased primer length, and shorter distance between primers. ITR sequences from GTPV and SPPV were targeted for LAMP primer design based upon previous bioinformatics analyses of CaPV genomes and corresponding homologs from other near-neighbor viruses listed in the NCBI (National Center for Biotechnology Information) database (data not shown). Functional LAMP primers were constructed (Table 1), with the nucleotide sequence of the target ITR amplicon and LAMP primer positioning depicted (Figure 1). LAMP amplicons (Figure 1) and LAMP primers (Table 1) were BLAST searched against the NCBI database to ensure their specificity. GTPV LAMP primers, F2, B2 and B3, were found to have 100% identity to the corresponding nucleotide sequences of the genomes of two GTPV isolates (GPV G20-LKV and GTPV Pellor), but none to other viral genomes. The SPPV LAMP primer, B2, was found to have 100% identity to the corresponding nucleotide sequences of the genomes of three SPPV isolates (SPV NISKHI, SPV A, and SPV 10700–99 strain TU VO2127), but none to other viral genomes. Additionally, GSPV LAMP primers were found to have 100% sequence identity to the genomes of several isolates of the CaPV genus, including three LSDV isolates (LSDV NI 2490, LSDV NWLW, and LSDV LW 1959). All primers exhibited no sequence identity to poxviruses outside the CaPV genus or to the host species sequences, including the caprine and ovine genomes (data not shown).

### Viruses and other pathogens

GTPV/SPPV nucleic acid samples extracted from GTPV or SPPV positive specimen were provided by the State Key Laboratory of Veterinary Etiological Biology (China CAAS). FMDV nucleic acid was provided by the national foot and mouth disease reference Laboratory, Lanzhou Veterinary Research Institute (LVRI) and *M. ovipneumoniae, Chlamydia psittaci, L. interrogans, Toxoplasma gondii, Theileria* and *Babesia sp* nucleic acid were provided by the Key Laboratory of Veterinary Parasitology of Gansu Province, LVRI, with samples stored at -80°C until further processing.

### DNA extraction

The clinical samples in the form of infected skin scabs, scrapes or nasal swabs were obtained from goats and sheep suspected of goat pox or sheep pox. Collections from different outbreaks were processed in a 10% (w/v) suspension in PBS (pH7.4), followed by DNA extraction utilizing a commercial DNA extraction kit per the manufacturer’s protocols (TaKaRa, Dalian, China).

### Reaction mixtures and optimal LAMP conditions

Initially, the LAMP assay was optimized by testing different concentrations of MgSO_4_, dNTPs and betaine, in conjunction with varying amplification temperatures (60-66°C) and reaction times (30, 45, 60 min) while using purified GTPV or SPPV gDNA (100 ng used in each reaction). Following optimization, the reaction was carried out in a volume of 25 μL containing 4.0 mM MgSO4, 1.4 mM dNTPs (each), 8 U/μL of Bst polymerase (large fragment; New England Biolabs, Sumido, Tokyo, Japan), 0.2% Tween 20, 10 mM KCl, 20 mM Tris–HCl, GSF3 and GSB3 primers (0.2 μM each), GSFIP and GSBIP primers (1.6 μM each) and 2 μL (100 ng) of extracted SPPV or GTPV gDNA. The amplification was performed in a water bath at 62°C for 60 min, followed by a 2 min incubation at 80°C to inactivate the Bst polymerase.

### Analysis of LAMP products

Two microliters of the LAMP products were separated electrophoretically in 2% agarose gel (Gelrose TM, Life Technologies, USA) containing 0.5 μg/ml ethidium bromide for 20 min at a constant 120 V. Results were visualized under ultraviolet (UV) light and images documented using a gel documentation system (Peiqing Image Biosystem, Shanghai, China).

### LAMP sensitivity

The sensitivity of the LAMP assay was tested using 10-fold serially diluted GTPV (1.045 × 10^9^ - 1.045 × 10^0^ copies per μL) or SPPV (1.037 × 10^9^ - 1.037 × 10^0^ copies per μL) genome as template.

### LAMP specificity

The specificity of the LAMP assays was confirmed using purified pathogen DNA or cDNA from sheep, goats and some wild ruminants to include GTPV, SPPV, Orf, FMDV, *M. ovipneumoniae, Chlamydia psittaci, L. interrogans, Toxoplasma gondii, Theileria* and *Babesia sp*, with a parallel negative control lacking template performed for each experimental set. To evaluate LAMP primer amplification specificity, amplified products were sequences by the Beijing Genomics Institute (BGI), Shenzhen, China and dataset were provided for bioinformatics analysis.

### Evaluation of the LAMP assay in clinical samples

RFLP-PCR analysis of the P32 and RPO30 gene in 135 preserved epidemic materials revealed 48 samples infected with goat pox and 87 infected with sheep pox [6,7]. Animal experimentation was performed inside the biosafety facilities of the Lanzhou Veterinary Research Institute, Chinese Academy of Agricultural Sciences (LVRI, CAAS), in compliance with the regulations of the Animal Ethics Procedures and Guidelines of the People’s Republic of China (AEPGPRC).

## Competing interests

The authors declare that they have no competing interests.

## Authors’ contributions

ZZ assisted in the experimental design of the study, wrote this manuscript and prepared the figures for publication. Most of the experimentation was conducted by BF who developed and optimized the LAMP assays, with GW who aided in partial plasmid construct and data analysis and XY who finished the RFLP-PCR experiments. QZ provided reference viruses and field isolates of GTPV and SPPV. The final manuscript was read and approved by all the authors.
